# Metabolomics of Genetically Modified Crops

**DOI:** 10.3390/ijms151018941

**Published:** 2014-10-20

**Authors:** Carolina Simó, Clara Ibáñez, Alberto Valdés, Alejandro Cifuentes, Virginia García-Cañas

**Affiliations:** Laboratory of Foodomics, Institute of Food Science Research (CIAL), Spanish National Research Council (CSIC), Nicolas Cabrera 9, Cantoblanco Campus, Madrid 28049, Spain; E-Mails: c.simo@csic.es (C.S.); clara.ibanez@csic.es (C.I.); a.valdes@csic.es (A.V.); a.cifuentes@csic.es (A.C.)

**Keywords:** metabolomics, transgenic, food, genetically modified organism, crops

## Abstract

Metabolomic-based approaches are increasingly applied to analyse genetically modified organisms (GMOs) making it possible to obtain broader and deeper information on the composition of GMOs compared to that obtained from traditional analytical approaches. The combination in metabolomics of advanced analytical methods and bioinformatics tools provides wide chemical compositional data that contributes to corroborate (or not) the substantial equivalence and occurrence of unintended changes resulting from genetic transformation. This review provides insight into recent progress in metabolomics studies on transgenic crops focusing mainly in papers published in the last decade.

## 1. Introduction

The application of genetic engineering is considered one of the leading technological advances in modern biotechnology. The organisms derived from genetic engineering are commonly named genetically modified organisms (GMOs). Since the production of the first genetically modified (GM) plant in 1983, a variety of agronomic traits that include benefits in agronomic productivity and industrial processing have been developed. Among the most relevant traits present in authorized GM crops, tolerance to herbicide and resistance to insects are prevalent worldwide. However, value-added traits such as different micronutrient content, faster ripening, improved feed value, and high levels of antioxidants, have also gained much attention recently [1,2,3].

Despite its important economic potential, authorization and commercialization of GMOs has been always controversial within the scientific community and the public sector. Several aspects of GMOs, including risk assessment, marketing, labeling, and traceability are strictly regulated in the European Union and other countries. In such regulations, the starting point in risk assessment of GMOs relies on the substantial equivalence concept that involves the comparison of the GMO under assessment with traditional varieties. Substantial equivalence concept is based on the assumption that commercialized traditional crops have been consumed for decades and have gained a history of safe use. Therefore, they can be used as comparators for the safety assessment of new GMOs derived from established plant varieties. One of the central safety issues under debate regarding GMOs is the occurrence of unintended changes resulting from genetic transformation. Unintended effects go beyond the primary expected effects of genetic modification, and represent statistically significant differences in a phenotype compared with an appropriate phenotype control [4]. Such unpredictable alterations are considered a significant source of uncertainty that might have an impact on human health and/or the environment [5].

Substantial equivalence evaluations are commonly approached using targeted analysis of predefined compounds that include natural toxins, macro-, micro-, and anti-nutrients, following recommendations in the Organization of Economic Cooperation and Development (OECD) consensus documents for individual crops [6]. This targeted approach has enabled the identification of unintended effects in some cases; however, its adoption within the substantial equivalence framework has raised several criticisms. Specifically, it has been argued that this targeted approach is biased, and that some unforeseen, unintended effects may escape detection [7]. In response to the bias and uncertainties associated with targeted analysis in comparative compositional evaluation of GMOs, a report by a panel of European Food Safety Agency recommended the development and use of profiling technologies such as omics technologies, with the potential to improve the breadth of comparative analyses [8]. More recently, a panel of experts on risk assessment and management has recommended profiling especially in cases where the most scientifically valid isogenic and conventional comparator would not grow, or not grow as well, under the relevant stress condition [9]. However, certain questions have been raised about the value of molecular profiling for GMO risk assessment [10]. Some arguments against profiling rely on the lack of validated procedures and the difficulty to interpret the differences observed between a certain GMO and its comparator. However, a number of reports demonstrating the suitability and applicability of different profiling approaches for comparative analysis of GMOs suggest good acceptance of these fast-evolving techniques by the scientific community.

Omics technologies are essential tools for understanding the response of organisms to genetic and environmental changes [11]. In this context, metabolomics has the potential to provide new dimensions to GMO analysis, allowing detection of the effects (intended or not) that might take place as a result of genetic engineering application. However, metabolomic analysis faces some challenges since the diversity of metabolites found in plants is by far greater than in other organisms, being the actual size of the plant metabolome unknown [12,13,14,15]. A group of well-established analytical techniques, namely, nuclear magnetic resonance (NMR) and mass spectrometry (MS)-based techniques, are the most commonly used in the vast majority of metabolic profiling and fingerprinting analyses of plants [16,17]. Although NMR requires limited sample preparation, medium to high abundance metabolites will be detected using this technique [18,19]. The field strength improvements in NMR superconducting magnets have increased the spectral resolution and detection sensitivity. Current MS-based approaches yield higher sensitivity than NMR when analyzing complex plant metabolite mixtures. The use of high and ultra-high resolution mass spectrometers greatly improves analytical performance and offers the best combination of selectivity and sensitivity [20,21]. However, to achieve the maximum high-throughput production of metabolic information from the analysis of the largest possible number of plant samples, sample pre-treatment should be reduced to a minimum. Moreover, a variety of novel direct MS-based approaches with great potential for metabolomics have been introduced in last years. An array of direct ionization or desorption/ionization techniques have been developed and combined for this purpose [22,23]. On the other side, the application of metabolomics approaches even to a limited number of samples results in a huge amount of data with its inherent difficulties in making a meaningful interpretation. In the last years, great efforts have been made to apply metabolomics approaches to investigate the compositional equivalence between GMOs and the conventional unmodified organisms. A variety of crops have been studied using mainly MS or NMR-based analytical platforms in combination with several statistical methodologies [4,24]. The most general approach to find meaning in metabolomics datasets involves the application of multivariate analysis methods such as for example, partial squares discriminant analysis (PLS-DA) and principal component analysis (PCA). Multivariate methods allow the identification of the spectral features contributing most to variation or separation for further analysis. PCA is one of the most frequently used unsupervised methods for metabolic fingerprinting and it provides a means to achieve unbiased dimensionality reduction. Unsupervised refers to the modeling being done without user intervention and only on the explanatory variables, leaving any responses optional for later stages in the process. However PCA only reveals group structure when within-group variation is sufficiently less than between group variation. On the other side, PLS-DA often performs more efficiently for the interpretable decomposition than PCA.

This review provides insight into recent progress in metabolomics studies on GM crops focusing mainly in papers published in the last decade (a list is given in Table 1). Below, cutting-edge applications of metabolomics in the context of GMO analysis are highlighted to illustrate its impressive potential.

**Table 1:** Metabolomic studies on GMOs (genetically modified organisms).

GM Crop	Tissue	Donor Specie	Genetic Modification	Phenotype	Analytical Technique	References
**Rice**	Seed	*B. thuringiensis*	*Cry1Ab*	Insect resistance	FTIR MS, NMR	[25]
	Seed	*B. thuringiensis*	*Cry1Ac, sck*	Insect resistance	GC-FID, GC-EI-Q MS	[26]
	Leaf	*Z. mays*	*C1, R-S*	Flavonoid production	LC-ESI-Q MS, LC-DAD	[27]
	Leaf, seed, root	*O. sativa*	*YK1*	Stress tolerance	CE-ESI-Q MS	[28]
	Seed	*O. sativa*	*RCH10, RAC22, β-Glu, B-RIP*	Antifungal activity	NIRS, GC-EI-Q MS, LC-DAD, ICP-AES	[29]
	Seed	*O. sativa*	*Mod. (Xa23, Xa21* genes)	Insect resistance	GC-EI-Q MS	[30]
	Seed	*B. thuringiensis*	*Cry1Ac, sck*	Insect Resistance	LC-ESI-Q/TOF MS	[31]
	Seed	*E. coli*	*GlgC-TM*	Nutritionally enhanced	LC-ESI-Q MS	[32]
	Seed	*N. tabacum*	*ASA2*	Nutritionally enhanced	LC-ESI-Q MS	[33]
	Seed	*A. tumefaciens*	*Psy-2A-CrtI Bar*	Nutritionally enhanced Herbicide tolerance	GC-EI-TOF MS	[34]
	Leaf, seed	*E. coli/O. sativa*	*LysC, dapA/LKR/SDH*	Nutritionally enhanced	LC-FTIR MS, GC-EI-Q MS	[35]
**Maize**	Grain	*B. thuringiensis*	*Cry1Ab*	Insect resistance	NMR	[36,37]
	Grain	*Z. mays*	*Mod. (Rpd3* gene)	Seed development	NMR	[38]
	Grain	*B. thuringiensis*	*Cry1Ab*	Insect resistance	NMR	[39]
	Grain	*B. thuringiensis*	*Cry1Ab*	Insect resistance	CE-ESI-TOF MS	[40]
	Grain	*B. thuringiensis*	*Cry1Ab*	Insect resistance	FT-ICR MS	[41]
	Grain	*B. thuringiensis*	*Bt toxin*	Insect resistance	GC-EI-Q MS	[42]
	Grain	B. thuringiensis	*Cry1Ab*	Insect resistance	GC-EI-Q MS	[43]
	Grain	*B. thuringiensis A. tumefaciens*	*Cry1Ab CP4 EPSPS*	Insect resistance Herbicide tolerance	GC-EI-Q MS	[44]
	Grain	*Z. mays*	*Mod. (Zmpsy1, Pacrtl, Gllycb, Glbch, ParacrtW* genes)	Nutritionally enhanced	LC-DA, LC-ESI-APCI MS	[45]
	Grain	*B. thuringiensis*	*Bt toxin*	Herbicide tolerance Insect resistance	NMR, GC-EI-Q-MS	[46]
**Soybean**	Seed	*A. tumefaciens*	*CP4 EPSPS*	Herbicide tolerance	GC-EI-Q MS	[42]
	Seed	*Agrobacterium spp.*	*837ASDIS*	Herbicide tolerance	GC-EI-Q MS	[43]
	Seed	*A. tumefaciens*	*CP4 EPSPS*	Herbicide tolerance	CE-ESI-TOF MS	[47]
	Leaf, EC, seed	*N. tabacum*	*ASA2*	Nutritionally enhanced	GC-EI-Q MS	[48]
	Seed	*A. tumefaciens*	*CP4 EPSPS*	Herbicide tolerance	CE-ESI-TOF MS	[49]
	Seed	*Avena spp*	*Mod. (HPPD* gene)	Herbicide tolerance	LC-ESI-Q MS, GC-EI-Q MS	[50]
	Seed	*A. tumefaciens*	*CP4 EPSPS*	Herbicide tolerance	CE-ESI-TOF MS, GC-EI-TOF MS, LC-ESI-Q/TOF MS, ICP MS	[51]
**Alfalfa**	Stem, leaf	*N. tabacum*	*PAL2*	Nutritionally enhanced	LC-UV	[52]
**Pea**	Leaf	*S. hygroscopicus*	*Bar*	Herbicide tolerance	NMR	[53]
**Wheat**	Leaf	*U. maydis*	*Chit/Gluc, RIP, Mod. (KP4* gene)	Fungal resistance	LC-DAD, LC-ESI-Q MS	[5]
	Seed	*T. aestivum*	*Glu-A1, Glu-D1*	Nutritionally enhanced	NMR	[54]
**Potato**	Tuber	*A. pullulans, S. tuberosum*	*W2, FK, Mal1, SamDC*	Starch biosynthesis, leaf morphology, ethylene production	GC-EI-Q MS	[6]
	Tuber	*S. tuberosum*	*AGPase, StcPGM, StpPGM*	Altered starch composition	GC-EI-Q MS	[55,56.^57^]
	Tuber	*C. scolymus*	*1-SST, 1-FFT*	Inulin synthesis	GC-EI-TOF MS, LC-ESI-Q MS	[58]
	Tuber	*A. tumefaciens*	*Potato virus Y*	Virus resistance	CE-ESI-IT-MS/MS	[59]
	Tuber	*A. thaliana*	*DREB1A*	Stress tolerance	GC-EI-TOF MS, LC-ESI-Q MS	[60]
	Tuber	*A. pullulans*	*W2*	Waxy phenotype	LC-UV, NMR	[61]
	Leaf	*S. cerevisiae*	*TPS1*	Drought resistance	GC-EI-Q MS	[62]
**Tomato**	Fruit	*A. tumafaciens*	*LBA4404*	Improved texture, mouthfeel, colour	NMR	[63]
	Leaf, fruit	*A. thaliana*	*AtHXK1*	Altered carbohydrate metabolism	GC-EI-Q MS	[64]
	Fruit	*Z. mays*	*LC1, C1*	Increased flavonol content	NMR	[65,66]
	Fruit	*E. coli*	*DXS*	Increased carotenoid content	LC-DAD	[67]
	Fruit	*V. vinifera*	*Stilbene synthase*	Resveratrol synthesis	LC-ESI-Q MS	[68]
	Fruit	*R. dulcifica*	Miraculin	Sweet flavor	GC-EI-TOF MS, LC-ESI-Q/TOF MS, CE-ESI/TOF MS	[69]
**Tobacco**	Leaf	*E. coli/P. fluorencens*	*Ent/CpmsB*	Salicylic acid producing plants	NMR	[70]
**Lettuce**	Leaf	*E. coli*	*Asn A*	Growth enhanced	NMR, GC-FID	[71,72,73]
**Cucumber**	Fruit	*T. daniellii*	*Thaumatin-II*	Sweet flavor	GC-EI-TOF MS	[74]
	Fruit	*T. daniellii*	*Preprothaumatin-II*	Aroma, sweet flavor	GC-EI-Q/TOF MS	[75]
**Raspberry**	Fruit	*RBDV*	*Virus movement protein*	Virus resistance	GC-EI-Q MS	[76]
**Grapevine**	Leaf	*E. coli*	*Adh*	Abiotic stress	GC-EI-Q MS, LC-ESI-IT MS, LC-DAD	[77]
**Peppermint**	Leaf	*Mentha x piperita*	*Mod. (MFS* gene), *DXR*	Essential oils content	GC-FID	[78]
**Cabbage**	Leaf	*A. tumafaciens*	*Bar*	Herbicide tolerance	LC-DAD, LC-ESI/Q MS	[79]
**Papaya**	Fruit	*C. papaya*	*Mod. (55-1* gene)	Virus resistance	LC-DAD, GC-FID	[80]
	Pulp, Leaf	*C. papaya*	*Mod. (rep* gene)	Virus resistance	GC-EI-Q MS, LC-DAD, LC-ESI-Q MS	[81]
**Poplar**	Cambial region	*P. trichocarpa*	*Mod. (hipI-SOD* gene)	Superoxide production	GC-EI-TOF MS, LC-ESI/TOF MS	[82]
**Barley**	Seed	*B. amyloliquefaciens*	*GluB, ChGP*	Antifungal activity	LC-ESI-IT MS	[83]

AES: Atomic Emission Spectroscopy; APCI: Atmospheric Pressure Chemical Ionization; CE: Capillary Electrophoresis; DAD: Diode Array Detector; EC: Embryogenic culture; EI: Electron Impact; ESI: Electrospray Ionization; FID: Flame Ionization Detector; FTIC: Fourier Transform Infrared Spectroscopy; GC: Gas Chromatography; ICP: Inductively Coupled Plasma; ICR: Ion Cyclotron Resonance; IT: Ion Trap; LC: Liquid Chromatography; *Mod.*: Modification; NIRS: Near Infrared Spectroscopy; Q: Quadrupole; *RBDV*: Raspberry bushy dwarf virus; TOF: Time Of Flight.

## 2. Metabolomics and GM (Genetically Modified) Crops: Case Studies

### 2.1. Rice

Rice (*Oryza sativa* L.) is one of the most important food crops in the world being the main source of calories and protein intake for half of the world population [84,85]. Significant advances have been performed in rice biotechnology in order to solve problems related to disease, insect, pest and abiotic stress (temperature, salt, nutrition, drought, wounding, *etc.*) that cause yield reduction. Metabolomics has proved to be a useful approach in the study of rice. Different technological developments in metabolomics applied to rice have been described [86], including their use in the determination of unexpected and undesirable compounds accumulated in GM rice.

GM rice (transformed with *cry1Ab* gene from *Bacillus thuringinesis*) and its wild type (WT) bred parent line were used as test materials to investigate the suitability of FTIR and NMR for metabolic fingerprinting, in combination with multivariate statistical analysis for sample classification [25]. The overall results indicated the advantage of supervised over unsupervised statistical analysis for classification purposes. The metabolic profiling of three insect-resistant GM rice lines with inserted *sck* (trypsin proteinase inhibitor derived from cowpea) and *cryIAc* transgenes was approached by Zhou *et al.* [26] using GC-FID. In that study, metabolic profiles of wild and GM rice varieties were compared to assess the unintended effects related to the genetic modification. In order to determine the environmental effects on metabolites, wild samples with different sowing dates or sites were analyzed. Results from that study indicated the levels of malic acid, asparagine, sorbitol and gluconic acid differed in rice planted at different locations whereas sucrose, mannitol and glutamic acid levels were the major metabolic differences affected by gene insertion. However, one of the main conclusions of that study was that growing conditions and gene modification induced similar influence on most of metabolites.

Amongst the numerous kinds of flavonoids produced in GM rice (transformed with maize *C1* and *R-S* regulatory genes), dihydroquercetin (taxifolin), dihydroisorhamnetin (3'-*O*-methyl taxifolin) and 3'-*O*-methyl quercetin were the major flavonoids detected using LC [27]. In a different report, Takahashi *et al.* [28] focused on the analysis of GM rice plants overexpressing NADPH-dependent HC-toxin reductase (*YK1*) gene product, which possesses dihydroflavonol-4-reductase (DFR) activity and provides biotic and abiotic stress tolerance. Authors applied CE-MS to analyze polar metabolites involved in glycolysis, tricarboxylic acid cycle and pentose phosphate pathways. The analyses indicated slight changes in the amounts of several metabolites in YK1-overexpressing plant tissues when compared with those of the same rice variety containing the hygromycin-resistant gene (vector alone). For instance, *cis*-aconitate, isocitrate and 2-oxoglutarate levels were higher in leaves, fructose-1,6-bisphosphate and glyceraldehyde-3-phosphate levels were lower in roots, and glutathione levels were significantly increased in calli. Although most of the metabolic changes could not be directly associated with the function of the transgene, authors hypothesized potential links between the elevation of glutathione level in calli, NADPH levels and DFR activity. Jiao *et al.* [29] reported a comprehensive metabolomics approach based on various analytical techniques including NIRS, GC-MS, LC, ICP-AES and chemometrics for the discrimination of three GM rice varieties from their conventional counterparts. The GM rice varieties included: (1) rice with resistance to blast, bearing four antifungal genes, *RCH10*, *RAC22*, *β-1,3-Glu*, and *B-RIP*; (2) rice with resistance to sheath blight, transformed to contain a rice chitinase gene, an alfalfa β-1,3-glucanase gene, and *p35H* containing a hygromycin phosphotransferase gene; and (3) rice with resistance to insects, containing *sck*, trypsin proteinase inhibitor derived from cowpea and *cry1Ac* gene from *Bacillus thuringiensis*. The levels of several amino acids, fatty acids, and vitamins, were altered to different extents in GM rice samples, suggesting that these unintended compositional alterations may be related to the genetic transformation. More recently, the metabolite profiles of a GM rice line C418-Xa21 (with bacterial blight-resistance genes), and two non-GM lines, C418 and C418/Xa23, were investigated using GC-MS [30]. After GC-MS analysis, cluster analysis (CA), PCA and PLS-DA were used to find differences between metabolic fingerprints obtained from different samples. As a conclusion of their study, authors indicated that the GM rice line was substantially equivalent to traditional cultivars apart from the change detected for succinic acid levels that fell outside the boundaries of natural variability observed between the two non-GM varieties. A similar approach, but using a different analytical platform, was adopted by Chang *et al.* [31] to investigate unintended effects of transgenic rice with *cry1Ac* and *sck* genes. In that work, LC-MS-based metabolomics in combination with PCA and PLS-DA were used to find the metabolites that permitted differentiating insect-resistant GM rice from its native counterparts. The authors also considered different sowing dates or locations as source of metabolite variation. Their findings suggested that environmental factors played a greater role than gene insertion for most metabolites. Although results also indicated slight variations in the levels of phytosphingosine, palmitic acid, 5-hydroxy-2-octadenoic acid and three other unidentified metabolites in the GM rice variety, the changes could not be related to the transgene.

The improvement of the nutritional properties in staple foods, such as rice, may have a major impact on the quality of life of the world’s population. The over-accumulation of primary or secondary metabolites caused by the introduction of the genetic modification may affect unexpected processes in the plant’s physiology through a sequence of several events that may include altered gene expression. Rice has been modified not only to improve agronomic traits but also to enhance its nutritional properties. The effect of the insertion of a cytoplasmic-localized *AGPase* mutant gene from *Escherichia coli* to enhance starch synthesis in rice was evaluated using LC-MS [32]. The studied GM rice lines showed elevated levels of ADP-glucose, accordingly with their higher AGPase activity. The levels of glucose 1-phosphate, UDP-glucose and glucose 6-phosphate were also elevated to the same relative extent in the GM lines compared with the WT rice line. A putative explanation for these changes was the inefficient utilization of ADP-glucose for starch synthesis due to a limitation in their transport into the amyloplast or as substrate by starch synthases. Glucose and fructose levels were also elevated in the GM rice. However, analysis of metabolite ratios showed no significant differences due to genetic manipulation. The same LC-MS analytical platform for metabolite profiling was applied to evaluate the additional effects of the tryptophan-fortified rice line by genetic engineering [33]. No marked effects on the amounts of other major metabolites were described in that work. However, uneven distribution of tryptophan in the plants was described in a time-dependent manner, with the highest level being observed in young developing tissues. Kim *et al.* [34] have recently studied the substantial equivalence between carotenoid fortified GM rice and five conventional rice cultivars (three white and two red grain colors) using GC-MS-based profiling of polar metabolites. It was suggested that GM rice was substantially equivalent to its conventional counterpart since GM rice was indistinguishable from the non-GM counterpart using the proposed non-targeted approach.

The GM-rice lines with increased lysine levels developed by Long *et al.* [35] are representative examples of essential nutrient improvement in crops by genetic engineering. It is noteworthy to mention that lysine is the first limiting essential amino acid in cereal grains (including rice). Authors genetically engineered rice to increase lysine levels following three different strategies: (1) expressing bacterial genes to enhance lysine biosynthesis; (2) using RNA interference of rice genes to down-regulate its catabolism; and (3) combining 1 and 2 to achieve both metabolic effects. The developed GM-rice plants contained free lysine levels increased up to ~12-fold in leaves and ~60-fold in seeds. In this work, the LC-MS technique was highly valuable for investigating the profile of 11 intermediate compounds involved in the Lys metabolic pathway in both rice seeds and leaves. These analyses revealed the existence of lysine catabolism in leaves and different regulatory mechanisms of lysine accumulation between rice leaves and seeds.

### 2.2. Maize

Maize (*Zea mays* L.) is another important crop worldwide for food, animal feed and bioenergy production [87]. GM maize, covering about 25% of total grown maize, has an important place in agriculture. Metabolomics has proved to be useful for predicting important agronomic traits. A first attempt to identify and classify maize seeds obtained from GM maize plants containing the *Cry1Ab* transgene, following a metabolomic fingerprinting approach, was carried out by Manetti *et al.* [36,37]. They demonstrated the capabilities of NMR and multivariate statistical data analysis to classify the maize seeds GM plants and their non-GM counterparts without the need of a complete assignment of the spectra. In another published report, a similar approach was used to study the introduction of the antisense-mediated down-regulation and over-expression of the *Rpd3* gene in the genome of a maize inbred line [38]. Piccioni *et al.* [39] also applied NMR to profile the metabolome of insect-resistant GM maize containing the *cry1A(b)* gene. Using this technique, 40 water-soluble metabolites were identified in all samples; nevertheless, based on the quantitative data, multivariate analysis allowed the discrimination between GM and non-GM samples. The metabolites responsible for such discrimination were ethanol, citric acid, glycine-betaine and trehalose, which showed higher levels in the GM maize samples. A clear link between the alterations in the concentration of these metabolites and the genetic modification was not found.

CE-MS has also proved to be helpful on the detection of the statistically significant differences in the metabolic profiles of varieties of conventional and insect-resistant GM maize [40]. Main differences were observed in the levels of l-carnitine and stachydrine between conventional and GM maize. The potential of combining two MS-based metabolomics approaches (namely, FTIR-MS and CE-MS) and pressurized liquid extraction (PLE), a green extraction technology, was evaluated later by the same research group to study GM maize [41]. Three GM varieties of insect-resistant maize and their corresponding isogenic lines grown under the same field conditions were analyzed. In that work, it was found that some metabolic pathways (amino acid, purine metabolism and folate biosynthesis, among others) were clearly altered in the GM varieties compared with their respective isogenic lines.

**Figure 1:**
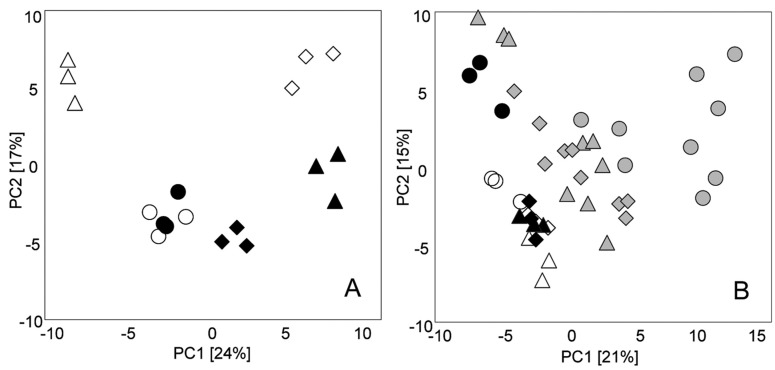
Principal component analysis of GC-MS metabolite profiling data (triplicate analysis of combined fractions I–IV) of Bt maize (∆, ▲), Roundup Ready maize (◊, ♦), and the near-isogenic counterpart (○, ●) grown at the locations Lichtenburg (white symbols) and Petit (black symbols) in 2004 (**A**) and at Petit in 2004 (○, ∆, ◊), 2005 (gray circle, gray triangle, gray diamond) and 2006 (●, ▲, ♦) (**B**). For Petit 2005, three field replicates were analyzed in triplicate. Reprinted with permission from Frank *et al.* [44], copyright 2012 American Chemical Society.

The amino acids profiles of insect-resistant GM maize (containing *cry1Ab* gene) and herbicide-tolerant soybean (containing CP4-EPSPS construct) were studied by GC-MS [42]. In that case, fast recovery of amino acids from maize and soybean grains was achieved with supercritical fluid extraction (SFE) with modified CO_2_. Following that procedure, various differences were detected in the amino acid profiles in GM maize and GM soybean when compared with their corresponding isogenic non-modified varieties. However, a direct association between the observed changes and the genetic modification could not be found. Following a similar analytical approach by GC-MS, the obtained profile of major and minor fatty acids in insect-resistant GM (*cry1Ab* gene) maize indicated high similarity when compared with its isogenic line grown in the same conditions [43]. On the contrary, the metabolic profiling by GC-MS carried out by Skogerson *et al.* [88] revealed that metabolome content was highly dependent on genotypic variation. More recently, Frank *et al.* [44] used also GC-MS profiling to investigate the impact of genetic modifications of insect-resistant maize (DKC78-15B, TXP 138F) and herbicide-tolerant maize (DKC78-35R) *versus* environmental influences (maize were grown together in different areas). The majority of differences observed were related to environmental factors rather than to the genetic modifications. Among them, location and season were predominant factors on the variability of metabolite profiles (Figure 1). Asiago *et al.* [89] designed a complex experiment to study the potential of metabolomic approaches to elucidate the biological variation in the expression of many metabolites due to environment, genotype, or both. Using GC-MS, 156 and 185 metabolites were measured in grain and forage maize samples, respectively. A similar approach, LC-MS was applied to detect a total of 286 and 857 metabolites in grain and forage samples, respectively [90]. The results indicated that the environment had the highest impact on the relative amounts of metabolites in both grain and forage. Rivera *et al.* [45] developed an ultra-high performance liquid chromatography (UHPLC)-MS method for carotenoid profiling to characterize GM maize lines expressing several carotenogenic genes, obtaining satisfactory results in terms of recoveries (82%–108%), detection limits (0.02–0.07 μg/mL) and repeatability (better than 13% Relative Standard Deviation, RSD) [45].

In general, studies are focused on acquiring only one omic level. Few attempts have been made to achieve comprehensive omic profiling in GM plants [82]. In this sense, Barros *et al.* [46] published an exploratory study about the use of different omics techniques to study the transcriptome, proteome and metabolome of two GM maize varieties (Bt and RR) for safety evaluation purposes. The effects of environmental conditions (year of harvest, agricultural practices and location) on GM maize were also studied at the three levels of information. Analysis using 1H-NMR and GC-MS platforms for metabolomics, gene expression microarray for transcriptomics and 2-D gel electrophoresis (2-DGE) analysis for proteomics revealed that the environment was shown to cause more variation in the gene, protein and metabolite expression of the maize samples than the different genotypes.

### 2.3. Soybean and Other Legumes

**Figure 2:**
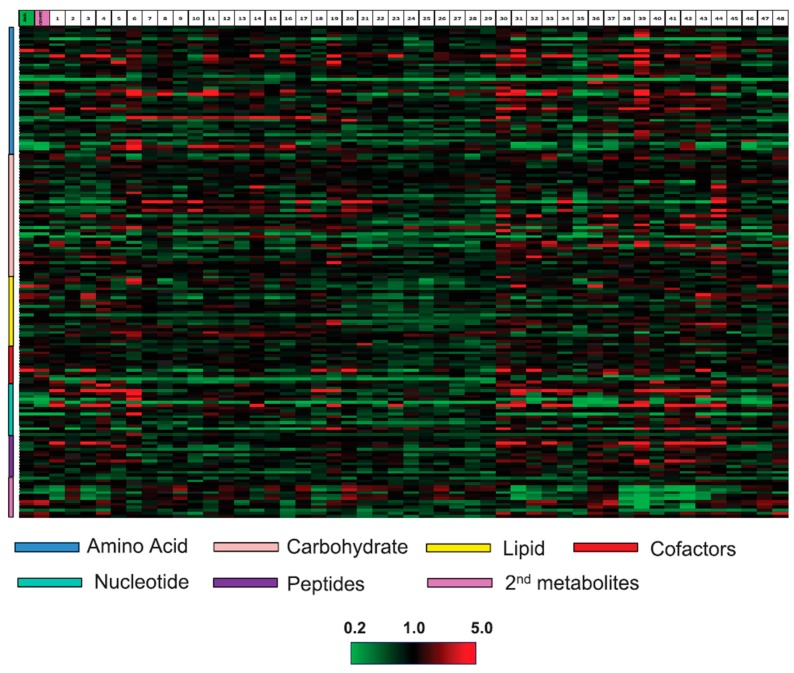
Metabolomic profiles and hierarchical clustering of 169 metabolites across the 49 soybean conventional lines and one GM line. The mean values for 8 biological replications per line were shown. Red and green indicate high and low levels, respectively, relative to the median value for all samples. The first (green label) and second (purple label) columns correspond to the isogenic and GM line, respectively. The columns numbered from 1 to 48 correspond to other conventional soybean cultivars. Reprinted with permission from Clarke *et al.* [50].

Soybean (*Glycine max*), classified under plant legume, is an important source of vegetable oil and protein. The GM soybean variety tolerant to glyphosate herbicide is one of the most extended GM crops in the world. The first work related to substantial equivalence of GM soybean through a metabolomic approach was carried out by García-Villalba *et al.* [47]. In that work, authors developed a CE-MS-based analytical strategy to compare the metabolic profile of conventional and GM soybean (*CP4 EPSPS* construct) [47]. More than 45 different metabolites, including carboxylic acids, isoflavones, and amino acids were tentatively identified. Among metabolite differences between GM and conventional soybean, most noteworthy differences were found in the concentration of proline, histidine, asparagine and 4-hydroxy-l-threonine. The latter, disappeared in the GM soybean compared to its parental non-GM line. In a comprehensive study, Inaba *et al.* [48] investigated the metabolite profile of herbicide-tolerant GM soybean, expressing an anthranilate synthase (*ASA2*) gene that is characterized by the accumulation of tryptophan in leaves, seeds and embryogenic cultures [48]. Metabolite profiles of different tissues were obtained by GC-MS revealing slight elevation of tyrosine and phenlylalanine levels in the GM soybean line. As authors mentioned in their work, a possible explanation for the elevation of these two amino acids in cells, that have increased levels of tryptophan, could be a feedback insensitive anthranilate synthase due to a mutation or transgene insertion, but further experiments are required to confirm this point. Also, Giuffrida *et al.* [49] studied the herbicide-tolerant GM soybean line using a chiral CE-MS method. Authors observed some quantitative differences in the chiral amino acid profile between the GM soybean and the untransformed genotype. However, further work remains to be done in order to investigate whether these differences could be a direct consequence of the genetic transformation. In a recent work, the issue of substantial equivalence assessment for herbicide-tolerant GM soybean has been addressed [50]. Thus, to study the metabolome within the context of the natural variation, 49 conventional soybean lines and one GM line were analyzed. Using LC-MS and GC-MS, the metabolome of the GM soybean presented no significant deviation from natural variation (represented in Figure 2) with the exception of changes in the targeted engineered pathway.

In a recent paper, Kusano *et al.* [51] have presented the results obtained from a comprehensive metabolomic study on a soybean lineage representing 35 years of breeding and increasing yield potential and three glyphosate-tolerant GM lines. The analytical strategy combined CE-MS, GC-MS, LC-MS and ICP-MS with multivariate analysis to successfully discriminate samples. The differences between the conventional and GM lines were small, indicating that genetic modification is not an important contributor to metabolite variability. Metabolomic data indicated that differences between older and newer soybean varieties provided novel and significant information on the impact of varietal development on biochemical variability, suggesting that safety assessments will need to consider that transgene insertion is not a major source of metabolite variability.

Apart from soybean, alfalfa (*Medicago sativa* L.) is a leading forage legume crop due to its high nutritive value. Several alfalfa lines have been genetically engineered to lower its lignin content with the final goal of improving alfalfa digestibility in ruminants. Thus, Chen *et al.* [52] used LC with UV detection to analyze soluble phenolics, wall-bound phenolics and cell wall lignins from GM alfalfa (with genetically downregulated *O*-methyltransferase genes) samples to study the effects of single gene disruption in the monolignol branch of phenylpropanoid biosynthesis. The results indicated that although the genetic modification decreased lignin biosynthesis, it also affected the levels of cell wall-bound ferulic acid. Pea plant, another well-known legume, has also been object of metabolomics studies. For instance, six independent lines of GM pea, transformed with a plasmid containing five transgenes and a *Ds* transposable element, were studied using NMR [53]. In their study, authors included the analysis of two control groups, the non-transformed pea plant control and the null segregant control from which the transgene has been lost. Multivariate analysis on the basis of PCA and linear discriminant analysis (LDA) failed to provide an acceptable classification of samples. In addition, statistical analysis revealed similar results, suggesting that the null segregant group was significantly different from the wild type, indicating that factors other than the presence of the transgene had significant effects on the metabolite profile.

### 2.4. Wheat

While much of the produced maize and soybeans are directed to feed animals or to be transformed into bioethanol, most wheat is consumed by humans as bread or pasta. Due to the socio-economic impact of this crop, GM wheat varieties have not legally been approved yet. Baker *et al.* [54] investigated the substantial equivalence of a series of GM wheat lines, transformed with *Glu-A1* and *Glu-D1* genes, with improved processing quality. In their study, the authors used NMR and multivariate analysis to compare the metabolite profiles of the GM wheat lines with their corresponding parental lines. The main differences between GM and parent lines were found in maltose and sucrose levels. However, a detailed explanation of the potential link between the observed metabolic changes and the genetic modification was not provided in that work. In the same study, GC-MS was employed for amino acid profiling, observing that differences between the control and GM lines were within the same range as the differences observed due to environmental factors (location and year).

### 2.5. Potato

**Figure 3:**
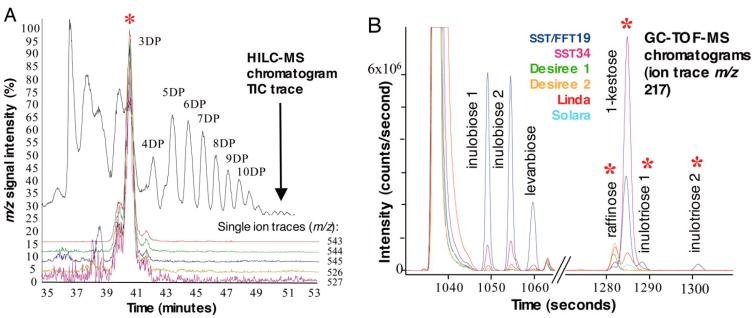
Identification of discriminatory metabolites in GM potato lines, some of them expressing *1-SST* and *1-FFT* (*SST/FFT*), and others expressing *1-SST* (*SST*), by LC-MS and GC-MS. (**A**) Overlaid single-ion chromatograms from LC-MS analysis of top-ranked predicted variables to represent ions derived from fructans, detected in *SST/FFT* potato tubers. Each color represents a single ion (*m*/*z*). Three degree of polymerization fructan is marked with a red asterisk; (**B**) GC-TOF extracted ion chromatogram *m*/*z* 217 for GM and non-GM potato tubers, enlarged for discriminatory disaccharide and trisaccharide regions. Separation of inulotriose 1 and inulotriose 2 from 1-kestose and raffinose is marked with a red asterisk. Metabolic signals from four conventional potato cultivars: Desiree 1 (green); Desiree 2 (yellow); Linda (red) and Solara (light blue), and two types of GM lines: SST34 (purple) and SST/FFT19 (dark blue) are represented in this figure. Reprinted with permission from Catchpole *et al.* [58].

Potato (*Solanum tuberosum* L.) is another relevant food crop in the world. It is also an alternative source of raw material for bioethanol production [91]. Potato has also been subject of a several metabolomic studies [92]. In a series of works, Roessner *et al.* [55,56,57] have demonstrated the potential of GC-MS for metabolite profiling of GM potato tubers. GC-MS was also the analytical platform of choice for the analysis of key compounds to investigate unintended effects in GM potato lines with altered carbohydrate metabolism (up-regulated or down-regulated fructokinase gene expression) [6]. The statistically significant differences between non-GM and GM lines were not associated with any specific construct. Interestingly, significant differences were also observed between non-transformed tubers and both, tissue culture derived tubers and tubers obtained from transformation with an empty vector, suggesting the occurrence of somaclonal variation. Global analysis of metabolite content using LC-MS and GC-MS was carried out to evaluate the degree of similarity between inulin-producing GM potatoes, transformed with *1-fructosyltransferase 1-SST*) and *fructan:fructan 1-fructosyltransferase 1-FFT*) genes, and conventional cultivars [58]. Multivariate analysis of chromatographic data showed that the most discriminatory ions with a significant impact on genotype separation were predicted to represent fructans of different degree of polymerization (DP), Figure 3.

Other relevant transgenic modification was based on the construction of virus resistant potato plants. Tubers were studied by CE-MS in order to determine and compare their glycoalkaloid content with equivalent non-GM potato variety [59]. CE-MS analysis revealed no substantial differences in GM tubers. Apart from targeted changes and the observed large variation in metabolite profile between the conventional cultivars, GM potatoes under study were shown to be substantially equivalent to traditional cultivars. In a recent work, differences between GM lines, containing dehydration response element-binding protein 1A transgene, and the non-GM parent cultivars were investigated [60]. In that work, LC-MS profiling revealed higher levels of the glutathione metabolite, γ-aminobutyric acid and β-cyanoalanine, a byproduct of ethylene biosynthesis, in the GM lines. Combinations of metabolic information provided by different analytical platforms (NMR and LC-UV) have been demonstrated to be largely complementary in terms of metabolites detected in potato tubers [61]. Information obtained from both analytical platforms was combined with the aim to detect unintended effects. Once more, it was found that the largest differences were found not between the GM potatoes and controls but between conventional varieties.

The responses to prolonged drought stress of wild type White Lady and the GM drought-tolerant TPS1 potato lines were studied by Kondrák *et al.* [62] at both transcriptional and metabolic levels. The expression of 57 genes was found to be altered in GM potato leaves compared to that in wild type potato leaves. Substantial increases in the detected proline, inositol and raffinose levels in the leaves of both potato lines seemed to be a general response to drought stress. In general, the biochemical changes detected did not clearly reflect the changes in gene expression. Authors concluded that inositol synthesis was influenced by transcriptional and/or biochemical changes induced, not exclusively by drought, but also by the transgene expression, whereas raffinose and proline synthesis were drought-specific.

### 2.6. Tomato

Tomato (*Lycopersicon esculentum*) is also a major food crop worldwide. The first work on metabolite comparison based on NMR analysis suggested minimal variations between isogenic non-GM and GM lines across sites or seasons (about 95% of all analyzed metabolites presented the same concentration) [63]. A metabolite profiling methodology, based on the combined use of GC-MS, conventional spectrophotometric LC and bioinformatic tools, was employed to study the influence of hexokinase activity on tomato fruit metabolism. To achieve this, different tissues of GM tomato plants overexpressing hexokinase gene product were analyzed at different developmental stages [64]. Their comprehensive analysis revealed some interesting findings regarding the influence of hexokinase on primary metabolism and its dependence on the environmental factors. A NMR study by Le Gall *et al.* [65,66] showed a significant increase of kaempferol glycosides in the flesh of GM tomato over-expressing maize transcription factors *LC* and *C1*. Apart from the significantly increased content of several flavonoid glycosides, the levels of other unrelated metabolites such as citric acid, sucrose, phenylalanine, and trigonelline, among others, were found to be different in GM tomato. However, the reported changes in mean values were relatively minor (less than 3-fold) and within the natural variation that would be observed in a field-grown crop. Long *et al.* [67] used LC to profile carotenoid and phenolic compounds of a panel of tomato lines representing a range of phenotypes of carotenoids or flavonoids levels (wild-type, mutants and various GM tomato lines). The GM lines included the *DXS* up-regulated plants which contained increased carotenoids, and the CRTI line, containing a bacterial (*Erwinia uredovora*) desaturase, which resulted in fruit with elevated (4.0-fold) β-carotene content and lutein levels and reduced (10-fold) phytoene levels. In the same investigation, tomato lines manipulated in cytochrome P450 ferulate 5-hydroxylase to increase the ferulate levels were also included. The study was aimed at investigating how manipulation of carotenoids or flavonoids pathways may affect other secondary metabolism pathways. It could be observed that perturbations to the biosynthesis of flavonoids or carotenoids independently did not affect the overall content of these relevant compounds. LC-MS was also employed for the analysis of GM tomato plants over-expressing grape stilbene synthase genes [68]. The analysis revealed that the genetic modification of the tomato plants originated from different levels of accumulation of four stilbenes (*i.e.*, *trans*- and *cis*-piceid and *trans-* and *cis*-resveratrol), depending on the stages of ripening. Other metabolites (rutin, naringenin, and chlorogenic acid) were suggested to be related to the genetic transformation. More recently, characterization of the GM tomato metabolome was approached by Kusano *et al.* [69]. In that study, two GM tomato varieties over-expressing miraculin glycoprotein and a panel of six traditional tomato cultivars were selected to prove a methodology based on three different metabolomics platforms. More specifically, data from CE-MS, GC-MS and LC-MS were summarized in single consensus datasets for further multivariate analysis. The combination of the three platforms allowed the statistical analysis of datasets containing over 175 unique tentatively identified metabolites and more than 1400 peaks with no or imprecise metabolite annotation. This analytical setup provided metabolite coverage of 85% of the chemical diversity found in the LycoCyc database. Results showed that >92% of the tested peaks in the transgenic lines deviated less from the control line than the accepted limit estimated using the reference panel of traditional cultivars.

### 2.7. Other GM Crops

GM tobacco has also been investigated in metabolomics studies. Choi *et al.* [70] applied 1H NMR and multivariate analysis techniques to differentiate wild type and GM tobacco plants overexpressing salicylate biosynthetic genes. The major compounds contributing to the discrimination were chlorogenic acid, malic acid, glucose and sucrose. Based on literature data, authors discussed potential associations between those altered metabolite levels and the increase of salicylic acid levels in plants; however, none of these changes in primary metabolites were directly related with salicylic acid biosynthesis. Sobolev *et al.* [71,72,73] published a series of papers where they applied NMR to investigate different metabolic aspects of GM lettuce with enhanced growth properties (over-expressing the asparagine synthetase A gene from *E. coli*). Statistical analysis of NMR data demonstrated significant increases in content of short-chain inulin oligosaccharides in GM lettuce leaves compared to those detected in the wild type plant. Interestingly, that was considered an unexpected effect because the transgenes aims at modifying the asparagine level, together with the nitrogen status, rather than the carbohydrate content [71,72,73].

An important application of transgenic modification is focused on the improvement of organoleptic characteristics of food. For instance, a GM cucumber plant has been transformed with the thaumatin-II gene to improve its taste [74]. The GC-MS analysis of five GM cucumber varieties bearing that transgene and its non-modified counterparts revealed that GM lines differ in their metabolic profiles and that those differences could be associated to the transgene integration site. However, the range of some of the observed changes was narrow, and authors classified them as somaclonal effects. Also, common changes in phenylalanine, aspartate, ethanolamine, pipecolate and benzoic acid levels were detected in the GM lines that could not be linked to the genetic modification. Another GM cucumber plant with enhanced aroma properties did not show significant differences when compared with its parental non-GM line by GC-MS [75]. Maolowicki *et al.* [76] studied the impact in certain metabolites in GM raspberry resistant to bushy dwarf virus (RBDV). Volatile compounds of RBDV-resistant raspberry lines were analyzed by GC-MS and compared with the wild-type. Whereas no flavor compounds tested in this study showed any difference between the GM lines and the wild-type raspberry, much larger variations were observed between sites and harvest seasons. The content of phenolic compounds and volatile secondary metabolites was investigated by GC-MS and LC-MS in GM grapevine plants over- and under-expressing alcohol dehydrogenase (*adh*) gene [77]. In that case, the main goal was to study the putative role of alcohol dehydrogenase in plant development and response to stress. Metabolite profiles indicated some differences in the degree of polymerization of proanthocyanidins, as well as increased levels of sucrose, carotenoid- and shikimate-derived volatiles in the GM plant. Supported by literature data, the authors discussed that some link could exist between alcohol dehydrogenase activity and the changes observed in sucrose metabolism. On the other side, the changes observed in secondary metabolites were not directly related with the role of alcohol dehydrogenase in the plant metabolism.

LC-DAD-based metabolic fingerprinting approach was used to evaluate undesirable changes in GM Chinese cabbage containing the *bar* gene [79]. In addition to genetic modification, sample periods (4- and 8-week old plants) were also subjected to study through metabolome analysis. It was observed that the time of samplings affected the metabolome in a higher extent than the genetic modification.

The advantages of using multiple analytical platforms to explore the composition of complex samples become evident. Adopting this strategy, complementary metabolomic information was obtained on the compositional differences between papaya (*Carica papaya* L.) transformed with the replicase gene for resistance to papaya ringspot virus (PRSV) and the non-GM counterpart [80,81]. Profiles of volatile organic compounds, sugar/polyals, organic acids, carotenoids and alkaloids in GM and non-GM papaya were obtained by GC-MS and LC-MS analytical platforms [81]. The metabolite variation between GM and non-GM papaya was slight during the two harvesting times studied in the work. Papayas harvested across different time periods showed a higher degree of compositional variability.

**Figure 4:**
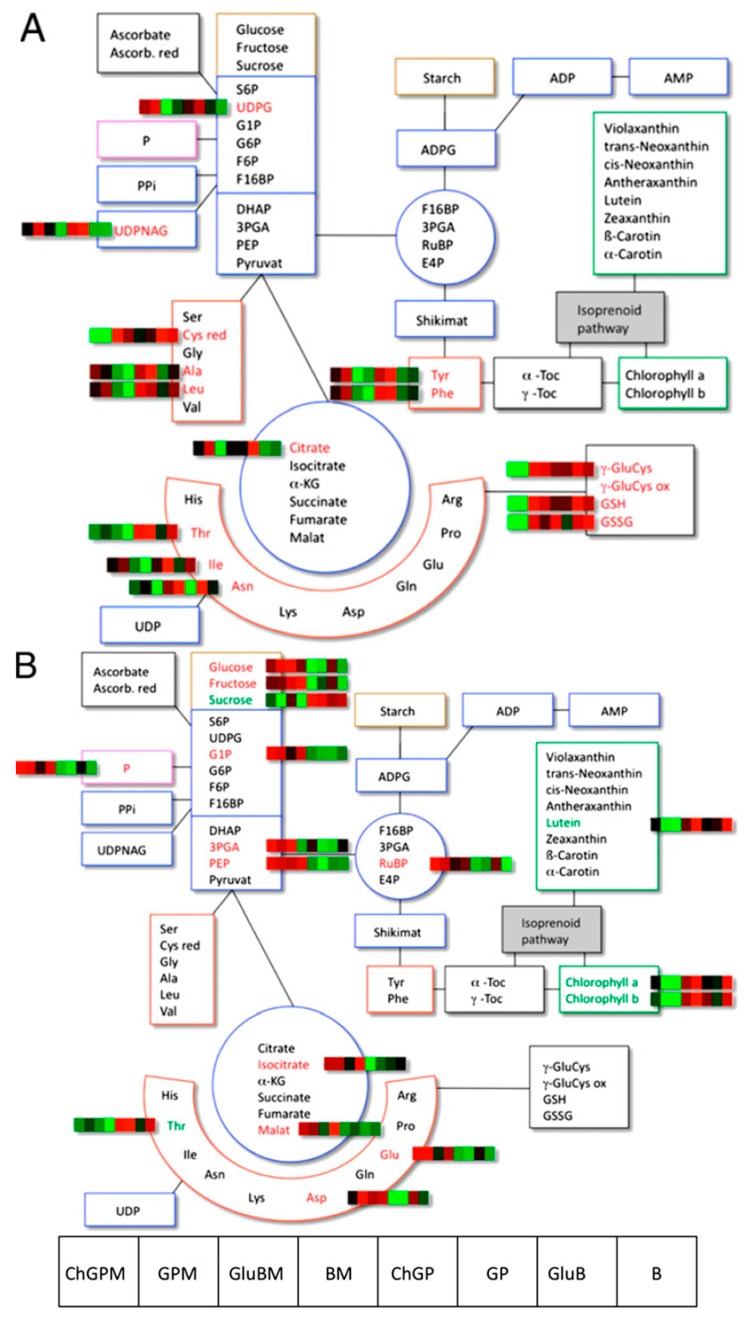
Differentially abundant metabolites in barley leaves. Overview of differentially abundant metabolites from the targeted profiling approach with leaf material from 4-month-old, field-grown barley plants representing the treatments (**A**) cultivar or (**B**) Amykor. The schematic metabolic diagrams in (**A**) and (**B**) represent a map of all analyzed metabolites. The heatmap strips next to the metabolite names were taken from the hierarchical cluster analysis, with red signals denoting an increased metabolite content relative to average and green signals indicating decreased metabolite contents relative to average. GP, Golden Promise; B, Baronesse; ChGP, Chitinase GP; GluB, Glucanase B; M, Amykor treatment. Reprinted with permission from Kogel *et al.* [83].

Transcriptome and metabolome analysis on GM barley plants (with different disease-resistance and nutritional traits) and their conventional counterparts grown in field with and without amendment of soil with mycorrhizal (Amykor) was performed by Kogel *et al.* [83]. In their work, a comparative analysis of 72 metabolites obtained in different culture conditions revealed slight differences in the abundance of some of them, as can be seen in Figure 4. The overall conclusion is that cultivar-specific differences in transcriptome and metabolome greatly exceed effects caused by transgene expression.

## 3. Concluding Remarks

A large number of new transgenic varieties of crops with desired traits are rapidly being introduced in the global market. The application of metabolomics for the safety assessment of GMOs is providing relevant information regarding the associated metabolite alteration as a result of gene modification. Exhaustive, unbiased metabolic profiling or fingerprinting of plants is greatly accelerating in the last years as demonstrated in this review. The chemical complexity of the plant metabolome, as well as the large dynamic range of concentrations are major challenges to be faced by metabolomics. To address these challenges, advances in analytical platforms have played a key role to unravel potential GM effects at the molecular level. Metabolomics studies (e.g., comparing GM crops with their wild type parent lines) are often combined with different culture conditions, from more geographical locations, multiple years, and different growing seasons, *etc.*, in order to investigate the natural metabolome variability. In general, results show that compared with genetic modifications, environmental variations usually produce greater major differences in metabolome composition.

Although the applications of metabolomics have not been validated yet within the regulatory framework for food safety assessment, it can be considered a powerful approach and a key strategy to screen compositional changes increasing the possibility of detecting unintended effects associated with genetic modification in GM plants. However, in order to understand the biological significance and impact of the detected changes, the large amount of data generated in metabolomics studies needs to be processed, integrated and interpreted together with data generated by other high-throughput technologies such as proteomics and transcriptomics as proposed by the new Foodomics strategy [93]. Although combinations of different omics technologies have been applied for the analysis of molecular alterations in GMOs, none of these studies have reported substantial correlations due to the lack of suitable integrative strategies. In this sense, the development of appropriate Systems Biology approaches providing new means to integrate and summarize omics data will help on the applicability of global approaches that can reveal new relationships, which cannot be found otherwise. However, before data integration procedures can be practically applied to GMO analysis, much effort will have to be made to develop effective integrative statistical approaches and appropriate computational frameworks for describing molecular systems and connecting omics databases. In this context, the development and implementation of initiatives to generate tools and databases that allow sharing metabolite data obtained from metabolomics studies will also be important [94].
